# U-shaped association between serum albumin and pediatric intensive care unit mortality in critically ill children

**DOI:** 10.3389/fnut.2022.931599

**Published:** 2022-08-30

**Authors:** Xuepeng Zhang, Lifan Zhang, Canzheng Wei, Liwei Feng, Juqin Yang, Geng Zhang, Guoyan Lu, Xiying Gui, Yue Zhou, Kaiying Yang, Jiangyuan Zhou, Xinle Zhou, Ruoran Wang, Siyuan Chen, Yi Ji

**Affiliations:** ^1^Department of Pediatric Surgery, West China Hospital of Sichuan University, Chengdu, China; ^2^Department of Critical Care Medicine, West China Hospital of Sichuan University, Chengdu, China; ^3^Department of Critical Care Medicine, Mianyang Central Hospital, University of Electronic Science and Technology of China, Mianyang, China; ^4^West China School of Medicine, Sichuan University, Chengdu, China; ^5^Department of Critical Care Medicine, The Second Affiliated Hospital of Shandong First Medical University, Tai’an, China; ^6^Biobank, West China Hospital, Sichuan University, Chengdu, China; ^7^Department of Pediatric Critical Care Medicine, West China Women’s and Children’s Hospital, Sichuan University, Chengdu, China; ^8^Department of Critical Care Medicine, People’s Hospital of Tibet Autonomous Region, Lhasa, China; ^9^Department of Pediatric Critical Care Medicine, Sichuan Provincial Maternity and Child Health Care Hospital, Chengdu, China

**Keywords:** children, albumin, critical care, restricted cubic spline, mortality

## Abstract

**Introduction:**

The detailed association between albumin levels and mortality has not been studied in critically ill children. The aim of this study was to reveal an association between albumin levels in detail and mortality in critically ill children.

**Materials and methods:**

We retrospectively collected data from children admitted to four pediatric intensive care units (PICUs) in China between January 2015 and October 2020. Restricted cubic spline curves based on logistic regression models were generated to evaluate the detailed associations between serum albumin levels and PICU mortality. Threshold effect analysis was performed using two piecewise regression models.

**Results:**

The study included 9,123 children. The overall mortality was 5.3%. The detailed association between serum albumin levels and the risk of mortality followed a U-shape. The risk of mortality decreased with increasing serum albumin levels (OR = 0.919; 95% CI: 0.886, 0.954) in children with serum albumin levels < 43.2 g/L and increased with increasing serum albumin levels (OR = 1.174; 95% CI: 1.044, 1.316) in children with serum albumin levels ≥ 43.2 g/L.

**Conclusion:**

There was a U-shaped association between serum albumin levels and mortality in critically ill children in the PICU.

A serum albumin level of less than 35 g/L is considered hypoalbuminemia, which is frequent in hospitalized patients, especially critically ill patients. It has been reported that more than 60% of pediatric patients have hypoalbuminemia in intensive care units. Many studies have revealed that hypoalbuminemia is associated with poor clinical outcomes. However, the detailed association between albumin levels and outcomes has not been well elucidated. In this study, we found a U-shaped association between serum albumin levels and mortality in critically ill children in the pediatric intensive care unit (PICU). Low albumin levels and high albumin levels were associated with mortality. The findings in this study suggest that maintaining the optimal serum albumin levels is important in critically ill patients in the PICU.

## Introduction

Serum albumin is the main contributor to intravascular colloid osmotic pressure. The normal range of serum albumin concentrations in healthy subjects is approximately 35–50 g/L. A serum albumin level of less than 35 g/L is considered hypoalbuminemia, which is frequent in hospitalized patients, especially critically ill patients. It has been reported that more than 60% of pediatric patients and nearly 50% of adult patients have hypoalbuminemia in intensive care units (ICUs) (1, 2). Albumin is frequently administered in the ICU to combat low albumin production. However, many albumin prescriptions are thought to be inappropriate, which may result in albumin administration being less cost-effective (3, 4). Determining the detailed association between albumin levels and outcomes could be clinically meaningful. On the one hand, it is impossible to maintain a serum albumin level above 35 g/L throughout the ICU stay. More importantly, this information could help intensivists appropriately administer albumin and reduce albumin abuse.

Serum albumin levels have long been recognized as an indicator of outcomes in hospitalized patients (5–8). A large number of studies have revealed that hypoalbuminemia is associated with adverse outcomes, including increased mortality, readmission, ICU admission, and prolonged hospital stay (1, 2, 8–10). However, the detailed association between albumin levels and outcomes has not been well elucidated. In this study, we aimed to quantify the association between serum albumin levels and mortality in critically ill children in the pediatric intensive care unit (PICU) in detail.

## Materials and methods

### Study population and setting

This was a retrospective study of critically ill children admitted to four PICUs in three tertiary referral hospitals in China. None of the four PICUs were a neonatal intensive care unit (NICU) and only received very few neonates. All children admitted to the PICUs between January 2015 and October 2020 were included in this study except those discharged from the PICU with uncertain outcomes, those without documented albumin values during the ICU stay, and those older than 18 years. The study was approved by the Ethics Committee of the Central Processing Center (West China Hospital of Sichuan University) (20201113-202022402017). The requirement for informed consent was waived due to the retrospective nature of the study and because the study did not divulge the patients’ private information.

The patients’ demographics, clinical characteristic data, laboratory values, vital signs upon admission to the ICU, including respiratory rate, blood pressure, heart rate and oxygen saturation, and PICU outcome were extracted from the Electronic Medical Record Systems of the hospitals. Albumin levels on admission, the lowest albumin level during the PICU stay, and the duration with an albumin level below the normal range were collected for analysis. The duration with an albumin level below the normal range was defined as the total number of days with an albumin level below 35 g/L during the PICU stay. It was 0 days for patients with no days with an albumin level below 35 g/L and 1 day for patients with a single day with albumin levels below 35 g/L. For patients with more than 1 day with an albumin level below 35 g/L, the duration was calculated by summing the number of days with an albumin level below 35 g/L. If there were days with albumin levels higher than 35 g/L between two albumin levels below 35 g/L, we separately calculated the durations and then added them together. Data management was performed by PostgreSQL version 11.11 (PostgreSQL Global Development Group).

### Statistics

The detailed associations between serum albumin levels and PICU mortality were evaluated with restricted cubic spline curves based on logistic regression models with adjustment for other covariates, including Pediatric Risk of Mortality (PRISM) score, age, race, sex, admission from emergency department, cardiac surgery, oxygen partial pressure (PO_2_), partial pressure of carbon dioxide (PCO_2_), shock index, respiratory rate (RR), bilirubin, estimated glomerular filtration rate (eGFR) being calculated according to Schwartz equation (11), mechanical ventilation, inotropes, and acute kidney injury (AKI) development (The baseline creatinine levels were the creatinine values on admission). The number of knots was set as 4 (0.05, 0.35, 0.65, 0.95) because 4 knots not only provide sufficient fit of the model but are a good compromise between flexibility and overfitting (12). Stratified analyses also used the same number of knots for comparison of overall and stratified analyses. The albumin levels not associated with mortality were defined as the albumin concentrations at which the 95% confidence interval (CI) of the odds ratio (OR) for mortality included a value of 1.0 in the cubic spline curves. Threshold effect analysis was performed using two piecewise regression models.

Continuous variables with normal distributions are presented as the means ± standard deviations (SDs). Non-normally distributed variables were described as medians [interquartile ranges (IQRs)]. Categorical variables were expressed as counts (percentages). *P* < 0.05 was considered statistically significant. All statistical analyses were performed in R version 3.6.1.

## Results

### Patient characteristics

A total of 9,671 pediatric patients were admitted to the PICU during the study period. Five hundred forty-eight patients were excluded for the following reasons: 235 patients had no albumin values during the ICU stay; 131 children had unknown outcomes; and 192 patients were older than 18 years. Finally, 9,123 patients were included in the analysis. Table 1 lists the characteristics of the patients by albumin centile subgroups according to the knots. In the overall population, the median age of the pediatric population was 1.0 (IQR, 0.0–4.0) years. The ratio of males was 54.7%. The mean albumin level was 38.2 ± 5.5 g/L. The most frequent reasons for PICU admission were respiratory disorders, followed by cardiovascular and digestive disorders. The median length of ICU stay was 3.0 (IQR, 1.0–6.0) days. The overall hospital mortality rate was 5.3%. The mortality of children with the lowest albumin level was 18.5%, which was the highest among the five categories.

**TABLE 1:** Baseline characteristics of the children.

	Albumin (g/L)					
	1st–5th <29.5 g/L *N* = 448	6th–35th 29.5–36.3 g/L *N* = 2,745	36th–65th 36.3–40.0 g/L *N* = 2,697	66th–95th 40.0–47.2 g/L *N* = 2,763	96th–100th ≥47.2 g/L *N* = 470	All *N* = 9,123
Albumin, g/L	26.0 (3.6)	33.6 (1.8)	38.1 (1.0)	42.6 (1.9)	49.9 (2.4)	38.2 (5.5)
Age, years	1 (0,6)	1 (0,4)	1 (0,4)	1 (0,4)	0 (0,2)	1 (0,4)
Albumin infusion, *n* (%)	146 (32.5%)	654 (23.8%)	467 (17.3%)	675 (24.4%)	96 (20.4%)	2,038 (22.3%)
Male, *n* (%)	250 (55.8%)	1,500 (54.6%)	1,476 (54.7%)	1,492 (54%)	271 (57.6%)	4,989 (54.7%)
**Race, *n* (%)**						
Han Tibet Yi Other	335 (74.8%) 53 (11.8%) 48 (10.7%) 12 (2.7%)	2,342 (85.3%) 199 (7.2%) 130 (4.7%) 74 (2.7%)	2,375 (88.1%) 145 (5.4%) 97 (3.6%) 80 (3.0%)	2,433 (88.1%) 158 (5.7%) 114 (4.1%) 58 (2.1%)	400 (85.1%) 32 (6.8%) 23 (4.9%) 15 (3.2%)	7,885 (86.4%) 587 (6.4%) 412 (4.5%) 239 (2.6%)
**Primary reason for PICU admission, *n* (%)**						
Respiratory	54 (12.0%)	909 (33.1%)	1,253 (46.5%)	1,143 (41.4%)	157 (33.4%)	3,516 (38.5%)
Cardiovascular	87 (19.4%)	581 (21.2%)	449 (16.6%)	527 (19.1%)	108 (23.1%)	1,752 (19.2%)
Digestive	112 (24.9%)	423 (15.4%)	372 (13.7%)	421 (15.3%)	96 (20.5%)	1,424 (15.6%)
Neurological	26 (5.8%)	196 (7.1%)	181 (6.7%)	178 (6.4%)	26 (5.5%)	607 (6.7%)
Malignant	60 (13.4%)	280 (10.2%)	195 (7.2%)	223 (8.1%)	23 (4.9%)	781 (8.6%)
Injury and poisoning	24 (5.3%)	78 (2.8%)	52 (1.9%)	49 (1.8%)	15 (3.2%)	218 (2.4%)
Other	86 (19.1%)	278 (10.1%)	195 (7.2%)	221 (7.9%)	45 (9.6%)	825 (9.1%)
Admission from emergency department	233 (52.0%)	784 (28.6%)	602 (22.3%)	736 (26.7%)	166 (35.3%)	2,521 (27.6%)
**Surgical type, *n* (%)**						
Non-cardiac surgery	190 (42.4%)	1,104 (40.2%)	1,014 (37.6%)	1,030 (37.3%)	176 (37.4%)	3,512 (38.5%)
Cardiac surgery	31 (6.9%)	471 (17.2%)	619 (22.9%)	588 (21.3%)	107 (22.8%)	1,816 (19.9%)
Non-surgical	227 (50.7%)	1,170 (42.6%)	1,064 (39.5%)	1,145 (41.4%)	187 (39.8%)	3,795 (41.6%)
Shock index	1.2 (0.9, 1.6)	1.2 (1.0, 1.5)	1.2 (1.0, 1.4)	1.2 (0.9, 1.4)	1.4 (1.1, 1.6)	1.2 (1.0, 1.5)
RR,/min	26 (20, 32)	26 (20, 30)	26 (20, 30)	28 (20, 31)	30 (23, 32)	26 (20, 30)
PO_2_, mmHg	85.9 (62.0, 100.0)	84.0 (65.4, 100.0)	89.7 (70.6, 102.6)	89.2 (70.0, 100.0)	87.0 (63.4, 100.0)	87.4 (68.2, 100.0)
PCO_2_, mmHg	40.1 (13.9)	39.7 (9.8)	40.2 (11.4)	39.6 (8.7)	42.7 (16.7)	40.1 (11.0)
eGFR, ml/min/1.73 m^2^	143.2 (82.8)	158.6 (65.0)	162.9 (57.0)	149.6 (58.6)	117.6 (47.4)	154.3 (61.2)
BUN, mmol/L	3.4 (2.3, 5.1)	3.3 (2.3, 4.7)	3.7 (2.7, 5.1)	4.0 (2.9, 5.4)	4.9 (3.6, 7.1)	3.7 (2.6, 5.1)
AKI, *n* (%)	164 (36.5%)	1,006 (36.6%)	1,008 (37.4%)	1,207 (43.7%)	267 (56.8%)	3,652 (40.0%)
Sodium, mmol/L	139.6 (6.6)	139.8 (4.8)	139.8 (4.5)	140.1 (6.1)	140.6 (4.8)	139.9 (4.8)
Hematocrit, %	0.3 (0.1)	0.3 (0.1)	0.3 (0.1)	0.4 (0.1)	0.4 (0.1)	0.3 (0.1)
Bilirubin, μmol/L	7.3 (3.2, 21.6)	8.0 (3.6, 16.2)	8.7 (4.3, 15.7)	9.7 (4.4, 18.1)	8.6 (4.3, 17.7)	8.6 (4.1, 16.9)
Inotropes, *n* (%)	18 (4.0%)	122 (4.4%)	110 (4.1%)	146 (5.3%)	44 (9.4%)	440 (4.8%)
Mechanical ventilation, *n* (%)	120 (26.7%)	713 (26.0%)	653 (24.2%)	662 (24.0%)	133 (28.3%)	2,281 (25.0%)
PRISM score	5.3 (3.5)	4.5 (2.4)	4.5 (2.4)	4.6 (2.6)	4.9 (3.2)	4.6 (2.6)
Mortality, *n* (%)	83 (18.5%)	145 (5.3%)	113 (4.2%)	106 (3.8%)	38 (8.1%)	485 (5.3%)
LOS, days	2 (1, 3)	3 (1, 5)	3 (1, 6)	4 (2, 8)	7 (4, 12)	3 (1, 6)

Values are presented as the mean (standard deviation), median (interquartile range) or number (%). PICU, Pediatric Intensive Care Unit; RR, Respiratory Rate; PO_2_, Oxygen Partial Pressure; PCO_2_, Partial Pressure of Carbon Dioxide; eGFR, estimated Glomerular Filtration Rate; BUN, Blood Urea Nitrogen; AKI, Acute Kidney Injury; PRISM Pediatric Risk of Mortality Score; LOS, Length Of PICU Stay.

### Association between serum albumin levels and mortality

**FIGURE 1:**
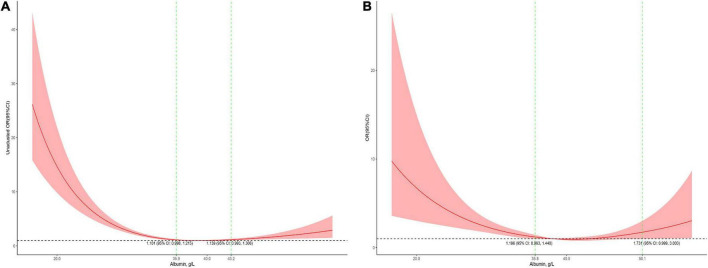
Association between serum albumin levels and mortality in all patients (**A**, the crude model; **B,** the model adjusted for age, race, sex, admission form emergency department, cardiac surgery, PO_2_, PCO_2_, shock index, RR, bilirubin, eGFR, mechanical ventilation, inotropes, AKI, and PRISM). Y-axis present the odds ratio, and the X-axis present albumin values. The 95% CI included an OR of 1.0 at albumin concentrations between 35.9 and 43.2 g/L in the crude model and between 35.9 and 50.1 g/L in the adjusted model.

**FIGURE 2:**
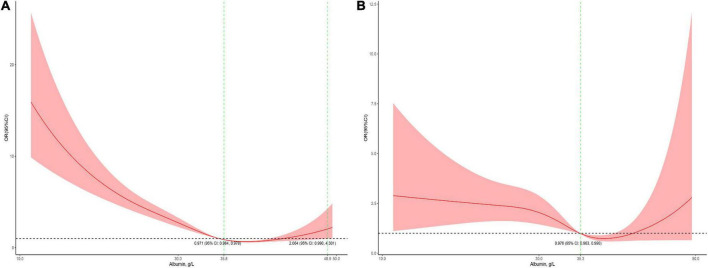
Association between the lowest serum albumin levels during the PICU stay and mortality in all patients (**A,** the crude model; **B,** the model adjusted for age, race, sex, admission form emergency department, cardiac surgery, PO_2_, PCO_2_, shock index, RR, bilirubin, eGFR, mechanical ventilation, inotropes, AKI, and PRISM). The 95% CI included an OR of 1.0 at albumin concentrations between 35.8 and 48.9 g/L in the crude model and > 35.3 in the adjusted model.

**FIGURE 3:**
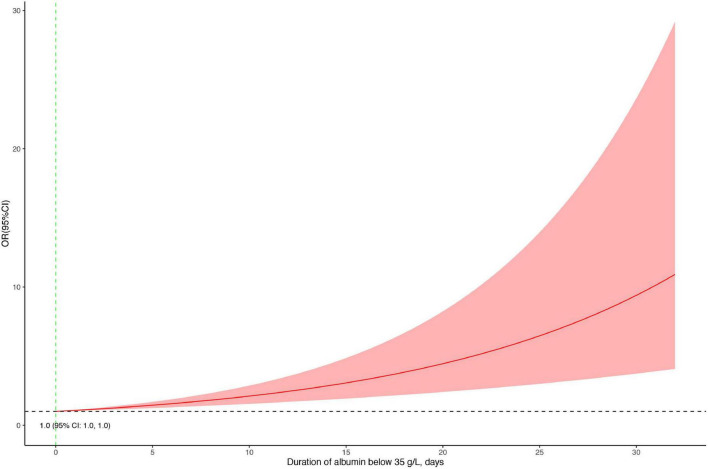
Association between the duration with an albumin level below 35 g/L and the risk of mortality.

The association between serum albumin levels and the risk of mortality was U-shaped (Figure 1). Low and high albumin levels were both associated with the risk of mortality. In the unadjusted cubic spline, albumin levels between 35.9 g/L (OR = 1.101; 95% CI: 0.998–1.215) and 43.2 g/L (OR = 1.139; 95% CI: 0.993–41.306) were not associated with mortality (Figure 1A). In multivariable adjusted analyses, the association between serum albumin levels and the risk of mortality still followed a U-shape (Figure 1B). Albumin levels between 35.8 g/L (OR = 1.196; 95% CI: 0.993–1.440) and 50.1 g/L (OR = 1.731; 95% CI: 0.999–3.0) were not associated with mortality in the adjusted cubic spline (Figure 1B). The albumin level associated with the lowest risk of mortality in multivariable analyses was 40.6 g/L. The lowest albumin levels also had a U-shaped association with the risk of mortality in the unadjusted model (Figure 2A) but not in the adjusted model (Figure 2B). In addition, the risk of mortality increased with increasing duration of albumin levels below 35 g/L (Figure 3).

### Threshold effect analysis

In the threshold effect analysis using two piecewise regression models, the turning points were 43.2 g/L in multivariable analyses and 35.8 g/L in univariable analysis (Table 2). In multivariable analyses, the risk of mortality was significantly decreased with the increment of albumin level (OR = 0.919; 95% CI: 0.886–0.954) in children with a serum albumin level < 43.2 g/L and increased with the increment of serum albumin level (OR = 1.174; 95% CI: 1.044–1.316) in children with a serum albumin level ≥ 43.2 g/L. In the univariable analysis, albumin level also showed a negative association with the risk of mortality (OR = 0.841; 95% CI: 0.815–0.867) in children with a serum albumin level < 35.8 g/L and a positive association with risk of mortality (OR = 1.038; 95% CI: 1.038–1.069) in children with a serum albumin level ≥ 35.8 g/L.

**TABLE 2:** Threshold effect analyses of serum albumin levels on mortality using two piecewise regression models.

	Adjusted			Crude	
Turnpoint	OR (95% CI)	P	Turnpoint	OR (95% CI)	*P*
<43.2 g/L	0.919 (0.886, 0.954)	<0.001	<35.8 g/L	0.841 (0.815, 0.867)	<0.001
≥43.2 g/L	1.174 (1.044, 1.316)	<0.01	≥35.8 g/L	1.038 (1.007, 1.069)	<0.05

OR, odds ratio; CI, confidence interval.

### Subgroup analysis

**FIGURE 4:**
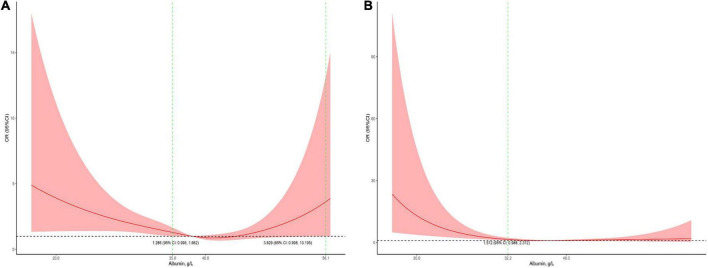
Stratified analysis for the association between serum albumin levels and mortality (**A,** subgroup analysis in male patients; **B**, subgroup analysis in female patients). The models were adjusted for age, race, admission form emergency department, cardiac surgery, PO_2_, PCO_2_, shock index, RR, bilirubin, eGFR, mechanical ventilation, inotropes, AKI, and PRISM.

**FIGURE 5:**
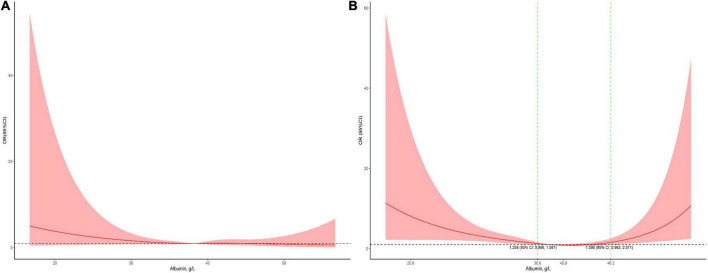
Stratified analysis for the association between serum albumin levels and mortality in surgical patients (**A,** subgroup analysis in cardiac surgery patients; **B,** subgroup analysis in non-cardiac surgery patients). The models were adjusted for age, race, sex, admission form emergency department, PO_2_, PCO_2_, shock index, RR, bilirubin, eGFR, mechanical ventilation, inotropes, AKI, and PRISM.

Serum albumin concentrations had a U-shaped association with the risk of mortality in male patients and a J-shaped association with the risk of mortality in female patients (Figure 4). In the children who received cardiac surgery, the 95% CI included an OR of 1.0 at any concentration of albumin (Figure 5A). In the patients who underwent non-cardiac surgery, the association between serum albumin and the risk of mortality followed a U-shape (Figure 5B).

## Discussion

In this study of 9,123 critically ill patients admitted to the PICU, we evaluated the association in detail between serum albumin concentrations and mortality by using restricted cubic spline curves based on a logistic regression model. We found a U-shaped association between serum albumin levels and the risk of mortality in critically ill children. Low and high albumin levels were both associated with a higher risk of mortality.

Serum albumin has been found to be a predictor of clinical outcomes in the last century (7, 13). A lower serum albumin concentration is inversely related to the risk of mortality. For each 25 g/L decrement in serum albumin concentration, the risk of mortality was estimated to increase as high as 56% (13). In addition to mortality, albumin levels have also been revealed to be associated with morbidities, such as sepsis and major infections (7). In a large prospective study, a decrease in albumin levels of 25 g/L was associated with an increase in morbidity of 55%. Consistently, albumin concentrations have also been shown to be associated with poor clinical outcomes in critically ill children (2, 14). Each increase of 10 g/L in serum albumin in critically ill children is associated with a 73% reduction in the risk of 60-day mortality (hazard ratio = 0.27; 95% CI: 0.14–0.51) (2). In a recent study, albumin levels still showed a negative association with mortality in patients admitted to the PICU (OR = 0.289; 95% CI: 0.136–0.615) (14).

In the current study, as expected, we also found an association between low serum albumin levels and a higher risk of mortality in critically ill children. In patients with serum albumin levels < 43.2 g/L, each increase of 1 g/L in serum albumin was associated with an 8.1% reduction in the risk of PICU mortality. This finding was similar to that of a previous study in critically ill children, in which a 7.3% reduction in the risk of mortality was associated with an increase of 1 g/L in serum albumin (hazard ratio = 0.27; 95% CI: 0.14–0.51) (2). In critically ill adult patients, each 1 g/L increase in serum albumin also resulted in a reduction in mortality of more than 60% (OR = 0.383; 95% CI: 0.198–0.740) (15).

What was new in this study was that high serum albumin also showed an association with mortality. There was a U-shaped association between serum albumin levels and the risk of mortality in critically ill children. To the best of our knowledge, there is no evidence that a high serum albumin level is associated with mortality in critically ill children. However, there was a study revealing a U-shaped association between clinical outcomes and children with end-stage renal disease (16). In the study, serum albumin levels < 35 g/L and ≥ 45 g/L were both associated with hospitalization frequency and hospitalization days. Similar findings were also found in adult patients. In a retrospective study, by stratifying patients into groups according to serum albumin levels, the investigators revealed that the risk of hospital-acquired AKI was related to a low albumin level (≤ 24 g/L) (OR = 1.52; 95% CI: 1.18–1.94) and a high albumin level (≥ 45 g/L) (OR = 2.16; 95% CI: 1.74–2.96) (17). In a recent study among adult patients with hypertension, a U-shaped association between serum albumin levels and chronic kidney disease was also revealed by using cubic spline curves (18). In the study, albumin levels < 51.4 g/L and ≥ 51.4 showed opposite associations with outcomes in patients (OR = 0.92; 95% CI: 0.88–0.96 vs. OR = 1.06; 95% CI: 1.01–1.11). The underlying mechanism of the association between high albumin levels and poor clinical outcomes is not fully understood. One of the potential mechanisms might be dehydration, which is common in sick young children. Studies have reported that high serum albumin levels are mostly caused by dehydration (19, 20). In the current study, the patients with the highest serum albumin levels had relatively higher blood urea nitrogen (BUN), hematocrit (HCT%) and serum sodium levels as well as a higher shock index but a lower estimated glomerular filtration rate (eGFR) (Table 1), which is a sign of dehydration. However, further studies are needed to understand the potential mechanism because high albumin levels are generally regarded as normal.

In children receiving cardiac surgery, hypoalbuminemia is associated with an increased length of hospital stay and mortality (21, 22). However, in this study, the serum albumin levels did not appear to be not associated with mortality in children receiving cardiac surgery. The most likely explanation may be that the number of patients with an albumin level < 29.5 g/L was too small. Only 31 patients undergoing cardiac surgery had albumin levels < 29.5 g/L, which may be too small to reveal statistical significance. Unlike the subgroup analysis in cardiac surgical patients, serum albumin levels showed the same U-shaped association with mortality in non-cardiac surgical patients.

In the adjusted analysis in the overall population, albumin levels between 35.8 and 50.1 g/L were not associated with mortality. This range might be clinically meaningful because maintaining the optimal serum albumin level is important in critically ill children in the PICU. Male patients had an approximate albumin level range between 35.6 and 56.1 g/L. The range in non-cardiac surgical patients was 36.6–46.2 g/L, narrower than those in the overall population.

This study has several limitations. First, although we included nearly all patients with documented albumin test results in this study, incomplete data were inevitable due to the inherent limitations of retrospective studies. Second, the dataset used for analysis came from large tertiary referral centers. The study design may limit the generalizability of our results to other non-tertiary centers and may introduce referral bias because of increased disease severity. Furthermore, we failed to reveal an association between serum albumin levels and mortality in children receiving cardiac surgery. This result should be cautiously treated because of the small sample of cardiac surgery patients with low albumin levels.

## Conclusion

In this study, we found a U-shaped association between serum albumin levels and mortality in critically ill children in the PICU. Both low and high albumin levels were associated with mortality. Serum albumin levels between 35.8 and 50.1 g/L were identified as the range not related to mortality in critically ill children. The range may suggest that maintaining optimal serum albumin levels is important in critically ill patients in the PICU.

## Data availability statement

The datasets used for the analysis in the current study are not in a public repository. However, the datasets are available from the corresponding author upon reasonable request.

## Ethics statement

The studies involving human participants were reviewed and approved by the Ethics Committee of the West China Hospital of Sichuan University. Written informed consent for participation was not provided by the participants’ legal guardians/next of kin because: The requirement for informed consent was not required due to the retrospective nature of the study and containing no individual information.

## Author contributions

XPZ, LZ, CW, SC, and YJ: concept and design. XPZ, LZ, CW, LF, GZ, KY, JZ, and RW: acquisition, analysis, and interpretation of data. XPZ, LZ, JY, and YZ: statistical analysis. LF, JY, and XG: administrative, technical, or material support. XPZ, CW, and LZ: drafting of the manuscript. SC and YJ: revision of the manuscript. All authors read and approved the final version of the manuscript.

## References

[1] van Beek DEC Königs MHH Kuijpers YAM van der Horst ICC Scheeren TWL. Predictive value of serum albumin levels on noradrenaline and fluid requirements in the first 24 h after admission to the intensive care unit – a prospective observational study. *J Crit Care.* (2018) 47:99–103. 10.1016/j.jcrc.2018.06.011 29940406

[2] Leite HP Rodrigues da Silva AV de Oliveira Iglesias SB Koch Nogueira PC. Serum albumin is an independent predictor of clinical outcomes in critically Ill children. *Pediatr Crit Care.* (2016) 17:e50–7. 10.1097/pcc.0000000000000596 26695729

[3] Caraceni P Domenicali M Tovoli A Napoli L Ricci CS Tufoni M Clinical indications for the albumin use: still a controversial issue. *Eur J Intern Med.* (2013) 24:721–8. 10.1016/j.ejim.2013.05.015 23790570

[4] Lyu PF Hockenberry JM Gaydos LM Howard DH Buchman TG Murphy DJ. Impact of a sequential intervention on albumin utilization in critical care. *Crit Care Med.* (2016) 44:1307–13. 10.1097/ccm.0000000000001638 26963324

[5] Reinhardt GF Myscofski JW Wilkens DB Dobrin PB Mangan JE Jr Stannard RT. Incidence and mortality of hypoalbuminemic patients in hospitalized veterans. *JPEN J Parenter Enteral Nutr.* (1980) 4:357–9. 10.1177/014860718000400404 6774116

[6] Ferguson RP O’Connor P Crabtree B Batchelor A Mitchell J Coppola D. Serum albumin and prealbumin as predictors of clinical outcomes of hospitalized elderly nursing home residents. *J Am Geriatr Soc.* (1993) 41:545–9. 10.1111/j.1532-5415.1993.tb01893.x 8486890

[7] Gibbs J Cull W Henderson W Daley J Hur K Khuri SF. Preoperative serum albumin level as a predictor of operative mortality and morbidity: results from the national VA surgical risk study. *Arch Surg.* (1999) 134:36–42. 10.1001/archsurg.134.1.36 9927128

[8] Viasus D Garcia-Vidal C Simonetti A Manresa F Dorca J Gudiol F Prognostic value of serum albumin levels in hospitalized adults with community-acquired pneumonia. *J Infect.* (2013) 66:415–23. 10.1016/j.jinf.2012.12.007 23286966

[9] Herrmann FR Safran C Levkoff SE Minaker KL. Serum albumin level on admission as a predictor of death, length of stay, and readmission. *Arch Intern Med.* (1992) 152:125–30. 1728907

[10] Rich MW Keller AJ Schechtman KB Marshall WG Jr Kouchoukos NT. Increased complications and prolonged hospital stay in elderly cardiac surgical patients with low serum albumin. *Am J Cardiol.* (1989) 63:714–8. 10.1016/0002-9149(89)90257-9 2923060

[11] Schwartz GJ Haycock GB Edelmann CM Jr Spitzer A. A simple estimate of glomerular filtration rate in children derived from body length and plasma creatinine. *Pediatrics.* (1976) 58:259–63. 951142

[12] Harrell FE. *Regression Modeling Strategies: With Applications to Linear Models, Logistic and Ordinal Regression, and Survival Analysis.* Berlin: Springer (2015).

[13] Goldwasser P Feldman J. Association of serum albumin and mortality risk. *J Clin Epidemiol.* (1997) 50:693–703. 10.1016/s0895-4356(97)00015-2 9250267

[14] Bekhit OE Yousef RM Abdelrasol HA Mohammed MA. Serum albumin level as a predictor of outcome in patients admitted to pediatric intensive care units. *Pediatr Emerg Care.* (2021) 37:e855–60. 10.1097/pec.0000000000002567 34908378

[15] Ramesh VJ Umamaheswara Rao GS Kandavel T Kumaraswamy SD Iyyamanda UB Chandramouli BA. Predictive model for survival among neurosurgical intensive care patients. *J Neurosurg Anesthesiol.* (2011) 23:183–7. 10.1097/ANA.0b013e31821cb9ec 21593685

[16] Okuda Y Obi Y Streja E Laster M Rhee C Langman CB Serum albumin and hospitalization among pediatric patients with end-stage renal disease who started dialysis therapy. *Pediatr Nephrol.* (2019) 34:1799–809. 10.1007/s00467-019-04270-2 31218394 PMC6776669

[17] Thongprayoon C Cheungpasitporn W Mao MA Sakhuja A Kashani K. U-shape association of serum albumin level and acute kidney injury risk in hospitalized patients. *PLoS One.* (2018) 13:e0199153. 10.1371/journal.pone.0199153 29927987 PMC6013099

[18] Jiang C Wang B Li Y Xie L Zhang X Wang J U-shaped association between serum albumin and development of chronic kidney disease in general hypertensive patients. *Clin Nutr.* (2020) 39:258–64. 10.1016/j.clnu.2019.02.002 30799192

[19] Wegman DH Apelqvist J Bottai M Ekström U García-Trabanino R Glaser J Intervention to diminish dehydration and kidney damage among sugarcane workers. *Scand J Work Environ Health.* (2018) 44:16–24. 10.5271/sjweh.3659 28691728

[20] Tanriverdi O. A discussion of serum albumin level in advanced-stage hepatocellular carcinoma: a medical oncologist’s perspective. *Med Oncol.* (2014) 31:282. 10.1007/s12032-014-0282-3 25316265

[21] Henry BM Borasino S Ortmann L Figueroa M Rahman A Hock KM Perioperative serum albumin and its influence on clinical outcomes in neonates and infants undergoing cardiac surgery with cardiopulmonary bypass: a multi-centre retrospective study. *Cardiol Young.* (2019) 29:761–7. 10.1017/s1047951119000738 31159896

[22] Leite HP Fisberg M de Carvalho WB de Camargo Carvalho AC. Serum albumin and clinical outcome in pediatric cardiac surgery. *Nutrition.* (2005) 21:553–8. 10.1016/j.nut.2004.08.026 15850960

